# Effects of a Plant-Based Diet During the First Month of Feeding on Alevin Rainbow Trout (*Oncorhynchus mykiss*) in the Development of Tongue Sensory System Regulating Feeding Behavior

**DOI:** 10.1155/anu/6690967

**Published:** 2025-04-26

**Authors:** Maud Martinat, Amelle Varvarais, Cécile Heraud, Anne Surget, Anthony Lanuque, Frederic Terrier, Jérôme Roy

**Affiliations:** Université de Pau et des Pays de l'Adour, INRAE, Aquapôle, NUMEA 64310, Saint-Pée-sur-Nivelle, France

**Keywords:** alevins, feeding behavior, first feeding, plant ingredients, tongue sensing system

## Abstract

Taste perception is essential for animals to detect nutrients, providing critical dietary information necessary for growth and survival. Since the early growth performance of alevin rainbow trout (*Oncorhynchus mykiss*) can be affected by food intake influenced by terrestrial ingredients without fish meal and fish oil, our study aimed to evaluate the role of taste receptors in nutrient detection and the associated signaling pathways leading to central nervous system activation in the regulation of feeding behavior. We conducted a nutritional experiment from the first feeding to 30 days, comparing the performance of fish fed a commercial-like diet (C diet: a blend of fish meal, fish oil, and plant ingredients) with those on a totally plant-based diet (V diet). After 5 and 30 days of feeding, fish were fasted for 16 h and then fed either the C or V diet, with sampling conducted at 20 min and 6 h post-meal. We evaluated the expression of nutrient-sensing genes related to fatty acids, amino acids, and sweetness, and taste receptor genes for flavors. Additionally, we examined calcium signaling pathways in the tongue, focusing on indolamine and catecholamine pathways, alongside appetite-regulating neuropeptides in the brain and intestinal hormones in the gut of alevins. Results indicated that fish on the V diet experienced a decrease in body weight gain starting 10 days after feeding to 30 days, along with changes in feed intake during the periods of 0–10 days and 21–30 days after the first meal. In tongue tissue, after 5 days of feeding, fish on the C diet showed a slight upregulation of nutrient taste receptors, but not those related to flavor, along with an upregulation of the calcium signaling pathway. By 30 days, there was a general upregulation of nutrient and flavor taste receptors, although the calcium signaling pathway showed less clear evidence of regulation. A significant dysregulation of the serotonin pathway, along with its degradation, was observed in the tongues of fish fed the V diet at both 5 and 30 days. For the first time in fish, catecholamine quantification levels in the tongue emerged as a potential marker for nutrient detection, with high quantification of L-DOPA after 5 days on the V diet, but much lower after 30 days. This impaired monoamine and catecholamine turnover in the tongue could be linked to a failure in activating the tongue-brain axis, potentially contributing to reduced food intake, as indicated by poorly regulated brain neuropeptides but also intestinal hormones in fish fed the V diet after 30 days. Overall, these findings demonstrate that the V diet disrupts the feeding response at an early stage, underscoring the heightened sensitivity of rainbow trout alevins' tongue sensing systems to novel food sources during critical early development.

## 1. Introduction

Aquaculture, as a rapidly growing sector, plays a crucial role in global food security. According to the FAO, [1] aquaculture production reached 114 million tonnes in 2022, accounting for ~46% of global fish consumption. However, the traditional aquaculture industry's reliance on marine ingredients, such as fishmeal (FM) and fish oil (FO), raises increasing ecological and economic concerns. In 2022, around 18 million tonnes of these marine ingredients were produced globally, with a significant portion used in farmed fish diets. This production requires the capture of large quantities of wild fish, often small species, which leads to the overexploitation of marine resources, threatening biodiversity and marine ecosystems. Moreover, the FAO [1] projects that the demand for aquaculture products will increase by 22% by 2030, further intensifying pressure on fish stocks. In response to these challenges, it is imperative to develop new diets that reduce or completely eliminate the use of FM and FO, which are considered the gold standard for farmed fish nutrition. Incorporating these alternatives into aquaculture diets not only reduces the environmental impact of the industry but also enhances the long-term resilience and sustainability of the sector. Innovation in feed formulations and research into the specific nutritional needs of aquaculture species are essential for this transition. In this way, aquaculture can evolve toward a more sustainable model, ensuring a reliable source of protein for future generations while preserving marine ecosystems [2]. Among aquaculture species, rainbow trout (*Oncorhynchus mykiss*) is a highly valued species, with a global production of ~275,000 tonnes per year. It is a significant source of protein, containing around 20%–25% protein and being rich in omega-3 fatty acids. Innovations in feed formulations can thus ensure optimal and sustainable growth for this specie, contributing to a more environmentally friendly aquaculture model [3]. Although adopting plant-based diets offers sustainability benefits, significant limitations have been observed regarding the growth performance of rainbow trout when fed total plant-based diets. Research shows that completely replacing FM/FO with plant ingredients can lead to a significant decrease in growth and survival during the early months of life [4–7]. As a result, the implementation of plant-based diets can result in economic losses and insufficient production to meet the growing demand for fish consumption. More recently, studies from our group, focusing on a long-term experiment (from first feeding to 8 months), have suggested that one possible explanation for the observed reduction in farming performance could be linked to impaired feeding behavior and a decreased preference for fish-free diets in rainbow trout [8, 9]. At the juvenile stage, following a single meal [10] or after 8 months on a total plant-based diet [5], defects in nutrient detection, along with altered systems regulating feeding behavior and reward [11], have been identified. Our data suggest that early feeding deficiencies could explain the long-term growth impairments. Thus, while the transition to total plant-based diets is promising for the sustainability of aquaculture, it remains essential to continue exploring the underlying biological mechanisms of nutrient detection to improve feed formulations. By understanding how these fish perceive and regulate nutrient intake, feed formulations can be better designed to optimize the use of available ingredients. A nutrient detection-based approach would allow for the creation of more effective feed formulations tailored to the specific needs of rainbow trout while incorporating a higher proportion of plant-based ingredients. In this study, we hypothesize that diets without FM/FO would alter the feeding behavior of rainbow trout alevins as early as the first month of life, following their first meal. This alteration would be linked to modifications in nutrient detection through the gustatory system (in tongue tissue), which would negatively impact feed intake and, in turn, lead to reduced farming performance that would persist over the long term. The complex processes that govern taste perception, and consequently the palatability of food, can undergo adaptations based on early life experiences, such as the acquisition of knowledge about food and its reward value [12, 13]. In fish, the gustatory system especially in tongue tissue plays a crucial role in detecting the taste of substances, which, in turn, triggers various feeding behaviors. Depending on how oral taste receptors perceive a substance, it can be classified as stimulating (palatable), aversive, or indifferent, influencing the likelihood of ingestion [14]. Numerous studies have investigated taste preferences and feeding behaviors in different fish species in response to various nutrients, compounds, or raw materials. These include fish farmed such as carp [15–17], Persian sturgeon [18], Nile tilapia [19–21], seabream [22–24] but also rainbow trout [8, 25–29], as well as a wide range of other fish species summarized in a review [30]. The signaling pathways involved in taste detection are also becoming well-documented in farmed fish, as summarized in this review [31]. However, while these studies are essential for understanding the mechanisms regulating feeding behavior, they predominantly focus on acute effects in juvenile fish and not on the critical early stages of feeding in alevins, a stage known to be crucial for feed acceptance. Thus, while the transition to FM/FO-free diets is promising for the sustainability of aquaculture, it is crucial to deepen our understanding of the biological mechanisms underlying nutrient detection. By identifying how rainbow trout perceive and regulate nutrient intake, it will be possible to improve feed formulations, optimizing the use of available ingredients. However, currently, little to no data are available on the mechanisms regulating feed intake in rainbow trout alevins. Consequently, this limits our ability to fully understand how the early development of the nutrient detection system in alevin trout may influence long-term phenotypic traits, which in turn could affect their feeding behavior and breeding performance. Moreover, a nutrient detection-based approach would enable the creation of more effective feed formulations tailored to the specific needs of rainbow trout while incorporating a larger proportion of plant-based ingredients. In this way, the aim of the present study was to evaluate the impact of the first month of feeding with a plant-based diet (referred as V diet), in comparison to a commercially formulated diet (containing FM, FO and plant ingredient sources, referred as C diet) on alevin trout nutrition detection mechanism. To achieve this, we conducted a nutritional experiment from the first feeding to 30 days, comparing the breeding parameters of fish fed with these C and V diets. After 5 and 30 days of feeding, fish were fasted for 16 h and then fed either the C or V diet, with sampling conducted at 20 min and 6 h post-meal. We investigated the expression patterns in the tongue of several nutrient receptors, including those for fatty acids, amino acids, carbohydrates, and flavor receptors (acid, bitter, sour, sweet, and umami). Additionally, we explored their associated calcium signaling pathways in the tongue and indolamine and catecholamine pathways. Then, we characterized the expression patterns of genes encoding neuropeptides that regulate appetite in the brain, as well as selected intestinal hormones in the gut that also influence feeding behavior. This comprehensive analysis of the critical first feeding stage in alevin trout will provide valuable insights into the regulatory mechanisms governing food detection in farmed fish.

## 2. Materials and Methods

### 2.1. Ethics Statement

The trial was conducted according to the guidelines of the National Legislation on Animal Care of the French Ministry of Research (decree no. 2013-118, 1 February 2013) and in accordance with European Union legal frameworks relating to the protection of animals used for scientific purposes (i.e., Directive 2010/63/EU). The trial was conducted at the INRAE's NuMeA facilities (https://doi.org/10.15454/GPYD-AM38) and approved by the ethical committee (C2EA-73) of INRAE, “Comité d'éthique Aquitaine poissons oiseaux” (INRAE agreement no. 21699, 19 December 2019). All efforts were made to minimize the number of fish used and their suffering. The scientists in charge of the trial received training and personal authorization.

### 2.2. Experimental Diets Design

Diets were manufactured at INRAE's experimental facilities at Donzacq (permit no. A40-228.1, Landes, France) using a twin-screw extruder (BC 45, Clextral, Firminy, France) after formulated using Allix software (A-Systems, City, France). The pellets had a diameter of 0.5/1 mm and a length of 0.5/1 mm. Details about the ingredients and composition of the experimental diets are given in Table 1 and the proportions of the main fatty acid and amino acid in the diets in Tables 2 and 3 respectively. The experiment was conducted with two different experimental diets: C diet containing a mix of FM (32%), FO (11.4%) and plant ingredients, and a V diet, completely free from FM and FO, which were replaced by a blend of plant ingredients 23.3% of soybean meal and 25.5% of peas and corn gluten to replace FM and 8% of palm oil and 4% of linseed oil to replace FO). This vegetal oil blend in V diet was chosen in order to achieve a similar proportion of fatty acid classes as found in C diet. For V diet, docosahexaenoic acid (DHA) and eicosapentaenoic acid (EPA) (present in FO for C diet) was replaced to the benefit of alpha-linolenic acid (ALA) by adding linseed oil (4%).

Diets were isoenergetic (*c*. 24.37 and 24.33 kJg^−1^ for C and V diet respectively) and were formulated to cover the nutritional requirements of the trout [32]. Diets were also isoproteic (53.4% ± 1.59% dry matter) and close to be isolipidic (18.82% ± 2.53% dry matter). The *ω*-3 polyunsaturated fatty acids (PUFAs) were present in comparable proportions in both diets. C diet contained 23.8% while the V diet had 26.6% *ω*-3 PUFAs, expressed as a percentage of total fatty acids. In terms of the overall composition of the diets, these percentages corresponded to 4.05% and 4.53% of the total diet, respectively, but consisted mainly of EPA, ALA, and DHA in the C diet and ALA in the V diet. This amount of *ω*-3 fatty acids (FAs) class was chosen in order to be close to the proportions of *ω*-3 FA classes found in the traditional diet of trout rich in FM/FO (more than 80% of aquafeed composition) [4]. In order to avoid exceeding antinutrient threshold levels, we used a blend of wheat gluten, soybean meal and whole wheat, corn gluten meal, soy protein, and peas as protein sources. L-lysine, L-methionine, dicalcium phosphate, and soy-lecithin were added to all diets to correct the deficiency in essential amino acids, phosphorous, and phospholipids. Mineral and vitamin premix were also added to each diet.

### 2.3. Proximate Composition

Nutrient compositions of the diets, crude protein, crude lipids, gross energy, ash content, and starch content, and fatty acid profiles were analyzed as previously described [5] and following the Official Methods of Analysis of AOAC INTERNATIONAL [33]. Briefly, the nutrient composition of the diets was analyzed after 24 h of drying at 105°C. Gross energy was determined using an adiabatic bomb calorimeter (IKA, Heitersheim Grißheimer, Germany). Ash content was measured by combustion in a muffle furnace (550°C for 8 h). Crude protein was quantified using the Kjeldahl method [34] and crude lipids using the Soxhlet method. Fatty acid profiles were measured gravimetrically according to the Folch method [35] using dichloromethane/methanol mixture (2/1).

### 2.4. Experimental Design

The female (genetically all female) alevin trout came from the same parental stock (INRAE Fish Farm of Lees-Athas, permit no. A64.104.1, Aspe Valley, France). Swimming fry with an initial body weight of 108 ± 0.1 mg were transferred in a raceway system at the INRAE's facilities of Donzacq (permit no. A40–228·1, Landes, France) for the feeding trial. Fingerlings were randomly allocated to five tanks per dietary treatment, with 150 fish per tank, this number to minimize the influence of tank size on feeding behavior. Water flow was set to ensure a dissolved oxygen concentration greater than 90% of saturation. Fish were exposed to natural photoperiod conditions and the water temperature was set at 17 ± 1°C. During the trial, the water conditions were as follows: dissolved oxygen was measured at 9 mgL^−1^, ammonia levels were below 0.01 mgL^−1^, nitrite levels were below 0.04 mgL^−1^, and nitrate concentration was approximately 17 ppm. The flow rate in each tank was 0.3 L/s, resulting in a complete water renewal of each tank 6 times per hour. During 30 days, all fish were fed by hand eight times a day with C or V diet, until apparent satiety. After 5 and 30 days, fish were starved during 16 h to obtain control condition (exclude feeding effect) and observe the postprandial regulation of feed intake by a meal. Afterwards, the trout were fed once either the C diet or the V diet.

**Figure 1:**
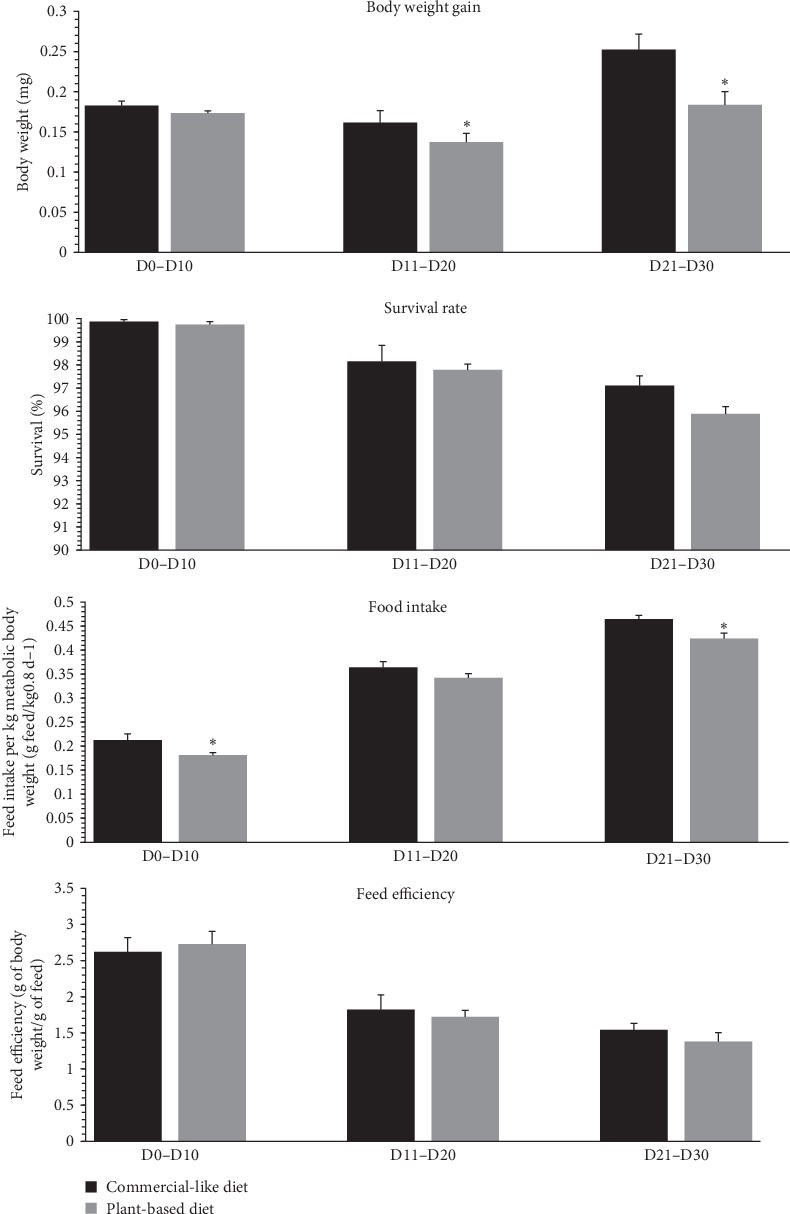
Husbandry parameters of trout fed a commercial-like (C) or plant-based (V) diet from first feeding to 1 month. Comparison of body weight gain (mg per periode of 10 days), survival rate (%), feed intake (g feed/kg^0.8^ d^−1^), and feed efficiency (g of BW/g of feed) for trout fed the C or V diet from 0–10, 11−20, 21–30 days after first feeding. An asterisk indicates a significant difference between the dietary treatments as determined by Welch Two Sample *t*-test (*p* < 0.05). Results are expressed in g as means ± SEM (*n* = 5 tanks).

At the end of the trial, fish biomass and the quantity of feed distributed per tank were measured by a period of 10 days to calculate mean body weight gain (mg per 10-day period) and survival rates (%), estimated daily food intake (in units per day), and estimated feed efficiency, as calculated below. For food intake, due to the difference in body weight between the two diets throughout the trial (Figure 1), these values were expressed per kg of metabolic body weight (MBW) per day. MBW was calculated as the geometric mean body weight gain (by period of 10 days), as previously described [5, 36].

 

  $$\begin{array}{c} \text{Growth}=\frac{Wf}{NBf} \end{array}$$

 

  $$\begin{array}{c} \text{Feed intake}=\frac{\text{Feed distributed}}{\frac{{\left(\frac{\text{Biomass}+Wi}{2}\right)}^{0.75}}{1000*10\left(\text{days}\right)}} \end{array}$$

  $$\begin{array}{c} \text{Feed efficiency}=\frac{\left(\text{Biomass}+Wd\right)-Wi}{\text{Feed distributed}} \end{array}$$

W_f_: Final weight for period

NB_f_: Final number of fish for period

W_i_: Initial weight for period

W_d_: Death weight for period

For tissue sampling, fish were first anesthetized by immersing them in a benzocaine solution (30 mg/L and euthanized in a bath containing 60 mg/L of benzocaine. Tongue, brain and gut were sampled before the single meal (16 h postprandial, pre-feeding) as well as 20 min and 6 h after the single meal. At this stage of rearing, whole tissues (non-zoned) were sampled to ensure reliable measurements and robust analytical conclusions. To minimize stress on the fish during sampling, we employed all five tanks per diet, thus avoiding the need to repeatedly sample the same tank 3 times for each timing point. The obtained tissues were subsequently immersed in liquid nitrogen to avoid degradation, and frozen at −80°C until mRNA or protein extraction.

### 2.5. Total RNA Isolation and Real-Time Quantitative PCR

Total RNA was isolated from the tongue, brain, and gut (*n* = 8 per dietary treatment) using the TRIzol reagent (Invitrogen, Carlsbad, CA, USA) with Precellys 24 (Bertin Technologies, Montigny-le-Bretonneux, France) in accordance with the instructions of the manufacturer, as previously described [37]. The quantities of extracted RNA were measured using a spectrophotometer (ND-1000, Nanodrop, Thermo), and the quality of extracted RNAs was estimated using the 260:280 nm OD ratio (purity of DNA and RNA) and 260:230 nm OD ratio (purity of solution, to avoid phenol contamination). For gene-expression analysis, only total RNA with a 260:280 nm OD ratio exceeding 1.8 and a 260:230 nm OD ratio exceeding 2.0 were utilized. Two micrograms of total RNA were reverse-transcribed into cDNA using the Super-Script III RNAse H-Reverse transcriptase kit (Invitrogen) and random primers (Promega, Chartonniéres-les-Bains) (Table 4) according to the manufacturer's recommendations. The LightCycler 480 system (Roche Diagnostics) was used to measure mRNA levels via real-time quantitative PCR (RT-qPCR). The reaction mixture was 6 μL and consisted of 2 μL of diluted cDNA (1:10), 0·24 μL of each primer (10 μmol/L), 3 μL of LightCycler 480 SYBR Green I Master mix and 0·52 μL of DNAse/RNAse-free water (5 Prime GmbH). Thermal cycling was initiated with incubation at 95°C for 10 min for the initial denaturation of the cDNA and hot-start Taq-polymerase activation, followed by 45 cycles of a two-step amplification program (15 s at 95°C; 10 s at 60°C). Cycle thresholds greater than 32 cycles were not considered. Melting curves were controlled systematically (temperature gradient: 1.1°C/15 s from 65°C to 97°C) at the end of the last amplification cycle to ensure that only one fragment was amplified. During a run, each sample was deposited twice, and two negative controls were added: a pool of samples without reverse transcriptase and a pool of samples without cDNA. The efficiency of all qPCR reactions was 96–100%. The sequences of amplicons had been previously assessed via sequencing [5, 10].

Data were extrapolated from standard curves and normalized to the housekeeping elongation factor 1 *α* gene (*eef1α*) for data from the brain and gut, and the keratin 8b gene (*krt8b*) for data from the tongue. Relative expression of the target genes was determined using the *ΔΔ*CT method [38]. The values for each group were expressed in fold changes relative to the T0 and presented as means ± 1 standard error of the mean (SEM) for all genes. Primer sequences used to amplify all paralog genes were previously validated and their accession numbers are presented in Table 4.

### 2.6. Indolamine and Catecholamine Release Measurement Using UHPLC-FL

Indolamine and catecholamine in the tongue (*n* = 10) from standard mixtures and polar metabolite extracts were separated using UHPLC (ACQUITY H-Class PLUS System, Waters, Milford, MA, USA), equipped with a thermostatted autosampler (4°C). Metabolites were detected through a fluorescence detector (ACQUITY FLR, Waters). Waters Empower Pro software was used to obtain and quantify the results.

All reagents and standards were purchased from Merck KGaA (Darmstadt, Germany). All organic solvents used were gradient MS grade (ADL & Prochilab, Lormont, France). Each tongue was homogenized with Precellys 24 (Bertin Technologies, Montigny-le-Bretonneux, France) in accordance with the instructions of the manufacturer, in 20 mM phosphate and 1 mM EDTA buffer (pH = 6.5 ± 0.05) with 1/5 (m/v) ratio. After the first centrifugation (14,000 × *g*, 20 min, 4°C), deproteinization of the supernatant was performed with a volume-to-volume 10% metaphosphoric acid solution. Following second centrifugation (14,000 × *g*, 5 min, 4 °C), the supernatant was filtered with a 0.22 µm poly-vinylidene di-fluoride unit through a third and last centrifugation (14,000 × *g*, 5 min, 4°C). Metabolite mixtures were stored at −20°C until analysis. Chromatographic separation was performed on an HPLC column (150 mm × 4.6 mm, i.d. 3 μm) at 30°C (Luna PFP (2), Phenomenex, Torrance, CA, USA). The injection volume was 10 μL, and the flow rate was set at 0.4 mL/min. A quaternary solvent system was used, which consisted of (A) a 10 mM phosphate buffer (pH 4.3 ± 0.05), (B) methanol, and (C) ultrapure water. The mobile phase was filtered through in-line 0.2 μm membrane filters. The following linear gradient elution was used: 0 min: 85% A, 15% B; 11 min: 80% A, 20% B; 11–14 min: 80% A, 20% B; 17 min: 70% A, 30% B; 17.1 min: 40% A, 60% B; 17.1–22 min: 40% A, 60% B; 22.1 min: 85% A, 15% C; 22.1–25 min: 85% A, 15% C; 25.1 min: 85% A, 15% B and 25.1–30 min: 85% A, 15% B. The eluate was monitored for identification and quantification using two sets of wavelengths. The first one, 285 nm (excitation)/355 nm (emission), was for indoles namely serotonin (5-HT) and 5-hydroxyindolacetic acid (5-HIAA). The second one, 280 nm (excitation)/330 nm (emission), was for catecholamines namely 3,4-dihydroxyphenylalanine (L-DOPA) and homovanillic acid (HVA). Metabolites of interest were identified by comparing retention times to standards. Quantification was based on integrating peak areas and comparing them to standard calibration curves (*R*^2^ > 0.995) of each metabolite of interest. Calibration curves were linear from 0.05 to 55 pmol per injection for each standard. Normalization was made using the total protein quantification using the bicinchoninic acid method (#BCA1-1KT, Sigma-Aldrich).

### 2.7. Statistical Analysis

**Figure 2:**
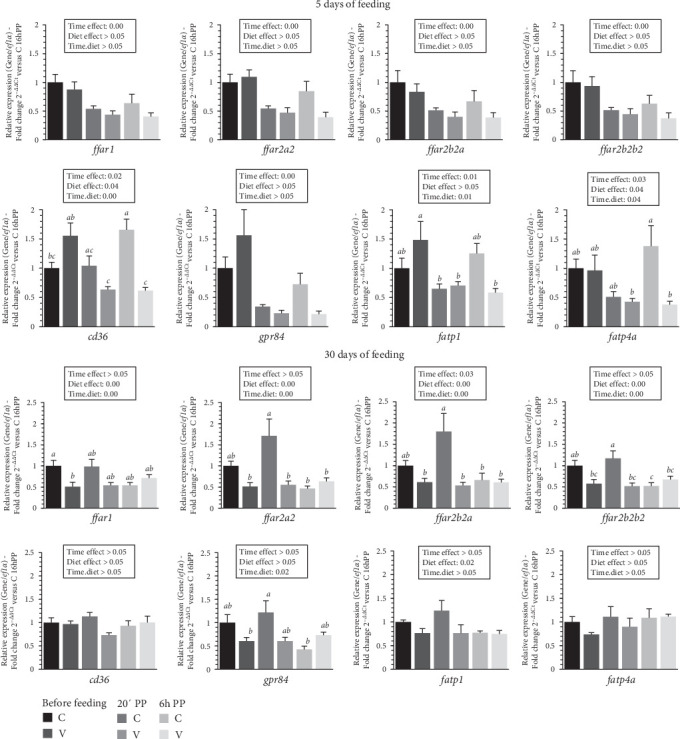
Levels of mRNA encoding genes of fatty acid receptors in the tongue tissue of trout fed C or V diet from first feeding to 5 days and 30 days. Relative gene expression measured by RT-PCR of selected fatty acid receptors (*ffar1*, *ffar2a2*, *ffar2b2a*, *ffar2b2b2*, *cd36*, *gpr84*, *fatp1*, *fatp4a*) genes of trout fed from first feeding to 5 days (top panel) and 30 days (bottom panel) the C or V diet before feeding, 20 min and 6 h after meal. Data are expressed as group mean ± SEM; fold change 2^–*ΔΔ*Ct^ versus before meal for all genes. Two-way ANOVA following by one-way ANOVA when the interaction is significant, Tukey's post hoc test when appropriate; different letters indicate a significant difference (*p*  < 0.05) between diet and time (*n* = 8).

**Figure 3:**
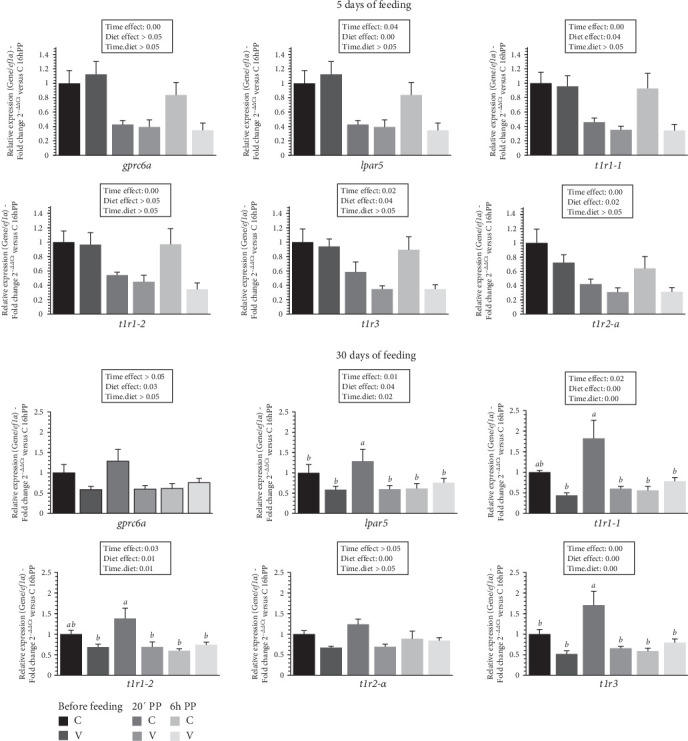
Levels of mRNA encoding genes of amino acid receptors in the tongue tissue of trout fed C or V diet from first feeding to 5 days and 30 days. Relative gene expression measured by RT-PCR of selected amino acid receptors (*gprc6a*, *lpar5*, *t1r1-1*, *t1r1-2*, *t1r2-a*, and *t1r3*) genes of trout fed from first feeding to 5 days (top panel) and 30 days (bottom panel) the C or V diet before feeding, 20 min and 6 h after meal. Data are expressed as group mean ± SEM; fold change 2^–*ΔΔ*Ct^ versus before meal for all genes. Two-way ANOVA following by one-way ANOVA when the interaction is significant, Tukey's post hoc test when appropriate; different letters indicate a significant difference (*p*  < 0.05) between diet and time (*n* = 8).

**Figure 4:**
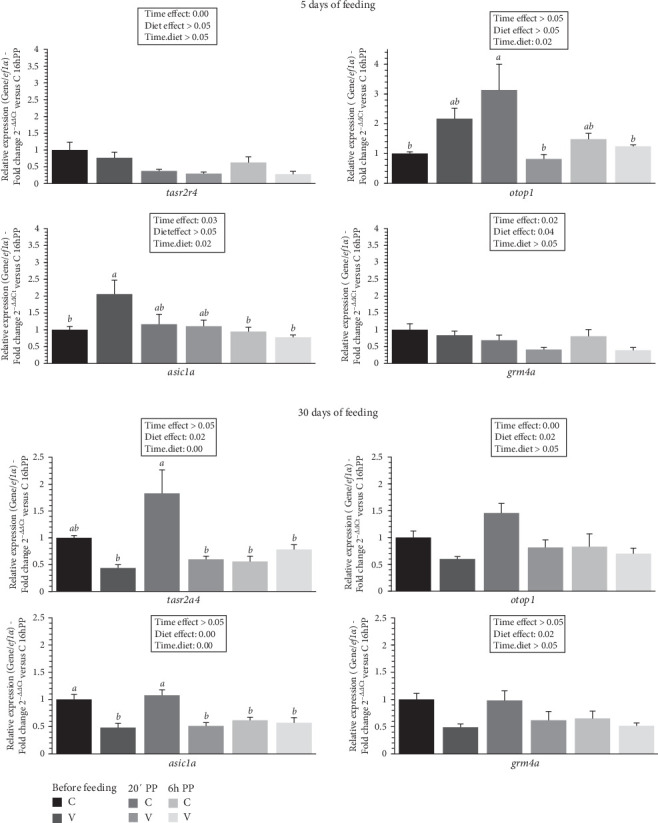
Levels of mRNA encoding genes of flavors receptors in the tongue tissue of trout fed C or V diet from first feeding to 5 days and 30 days. Relative gene expression measured by RT-PCR of selected flavors (*tas2r4*, *otop1*, *asic1a*, *gmr4a*) genes of trout fed from first feeding to 5 days (top panel) and 30 days (bottom panel) the C or V diet before feeding, 20 min and 6 h after meal. Data are expressed as group mean ± SEM; fold change 2^–*ΔΔ*Ct^ versus before meal for all genes. Two-way ANOVA following by one-way ANOVA when the interaction is significant, Tukey's post hoc test when appropriate; different letters indicate a significant difference (*p* < 0.05) between diet and time (*n* = 8).

**Figure 5:**
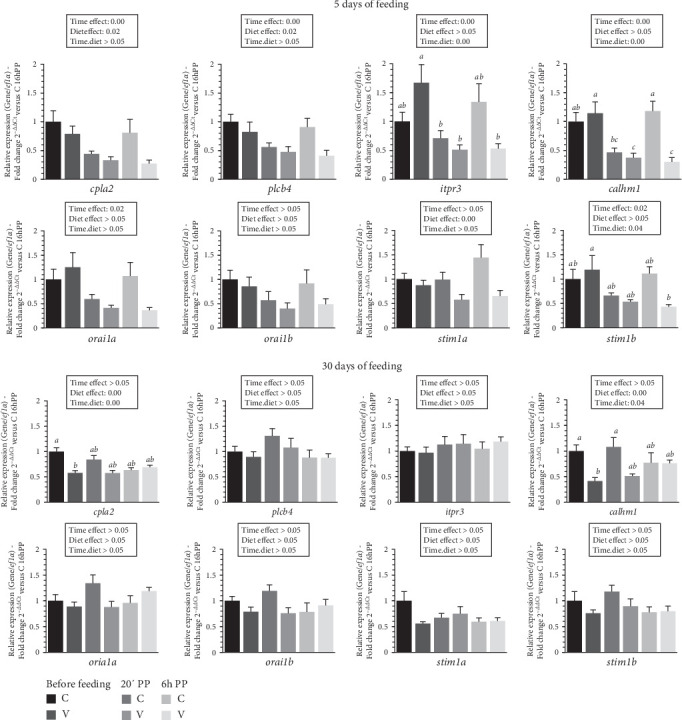
Levels of mRNA encoding genes of calcium-signaling pathways in the tongue tissue of trout fed C or V diet from first feeding to 5 days and 30 days. Relative gene expression measured by RT-PCR of selected calcium-signaling (*cpla2*, *plcb4*, *itpr3*, *calhm1*, *orai1a*, *orai1b*, *stim1a*, *stim1b*) genes of trout fed from first feeding to 5 days (top panel) and 30 days (bottom panel) the C or V diet before feeding, 20 min and 6 h after meal. Data are expressed as group mean ± SEM; fold change 2^–*ΔΔ*Ct^ versus before meal for all genes. Two-way ANOVA following by one-way ANOVA when the interaction is significant, Tukey's post hoc test when appropriate; different letters indicate a significant difference (*p* < 0.05) between diet and time (*n* = 8).

**Figure 6:**
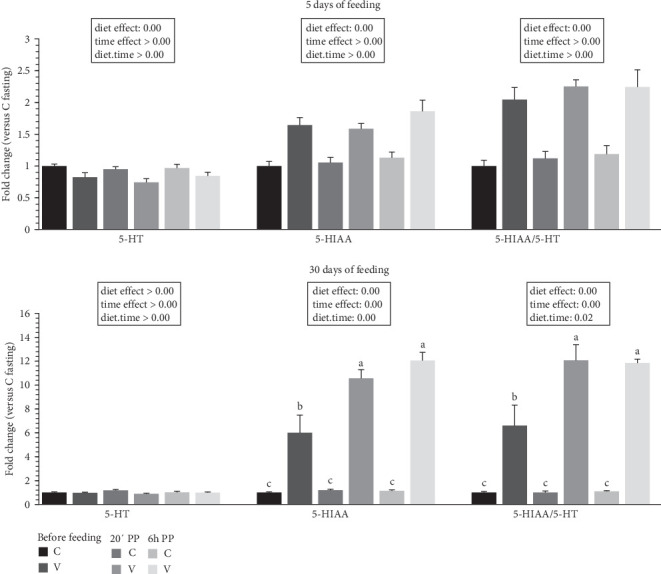
Indolamine metabolites turnover in tongue tissue of trout fed C or V diet from first feeding to 5 days and 30 days. Tongue concentration of indolamines (5-HT, 5-HIAA and turnover ratio 5-HT/5-HIAA) of trout fed from first feeding to 5 days (top panel) and 30 days (bottom panel) the C or V diet before feeding, 20 min and 6 h after meal. Data are shown as means ± SEM; fold change versus before meal (T0). Two-way ANOVA following by one-way ANOVA when the interaction is significant, Tukey's post hoc test when appropriate; different letters indicate a significant difference (*p*  < 0.05) between diet and time (*n* = 10).

**Figure 7:**
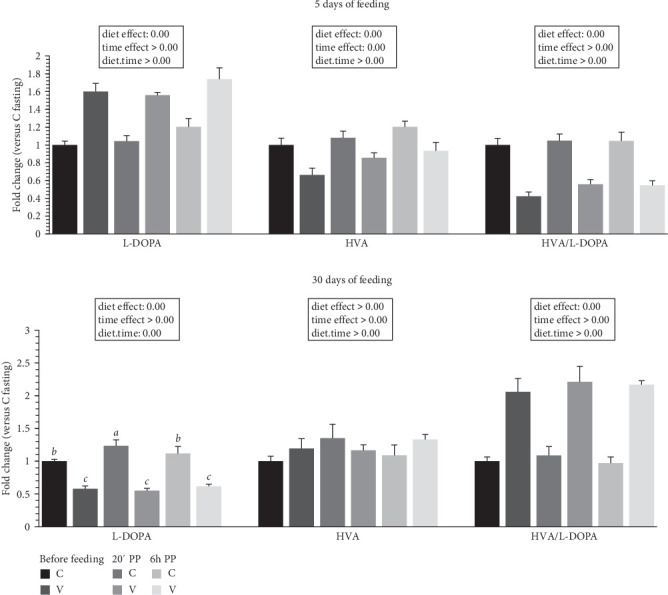
Catecholamine metabolites turnover in tongue tissue of trout fed C or V diet from first feeding to 5 days and 30 days. Tongue concentration of catecholamine (L-DOPA, HVA, and turnover ratio HVA/L-DOPA) of trout fed from first feeding to 5 days (top panel) and 30 days (bottom panel) the C or V diet before feeding, 20 min, and 6 h after meal. Data are shown as means ± SEM; fold change versus before meal (T0). Two-way ANOVA following by one-way ANOVA when the interaction is significant, Tukey's post hoc test when appropriate; different letters indicate a significant difference (*p*  < 0.05) between diet and time (*n* = 10).

**Figure 8:**
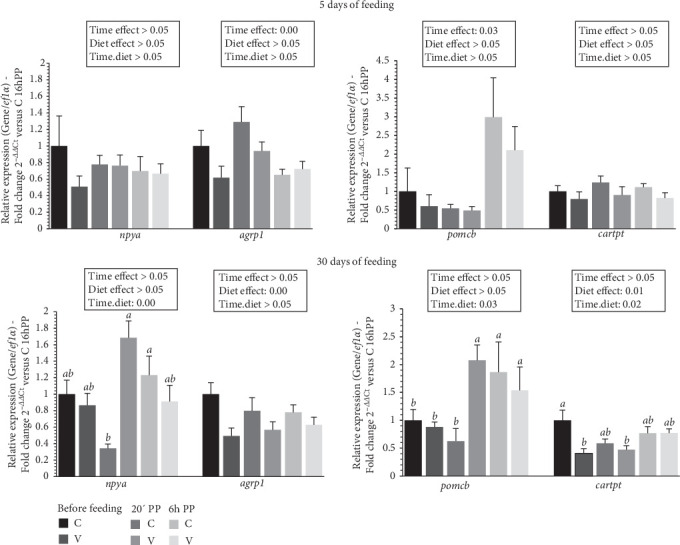
Levels of mRNA encoding genes of food intake markers in the brain tissue of trout fed C or V diet from first feeding to 5 days and 30 days. Relative gene expression measured by RT-PCR of selected food intake markers (*npya*, *agpr1*, *pomca*, *pomcb*, and *cartpt*) genes in brain tissue of trout fed from first feeding to 5 days (top panel) and 30 days (bottom panel) the C or V diet before feeding, 20 min, and 6 h after meal. Data are expressed as group mean ± SEM; fold change 2^–*ΔΔ*Ct^ versus before meal for all genes. Two-way ANOVA following by one-way ANOVA when the interaction is significant, Tukey's post hoc test when appropriate; different letters indicate a significant difference (*p*  < 0.05) between diet and time (*n* = 8).

**Figure 9:**
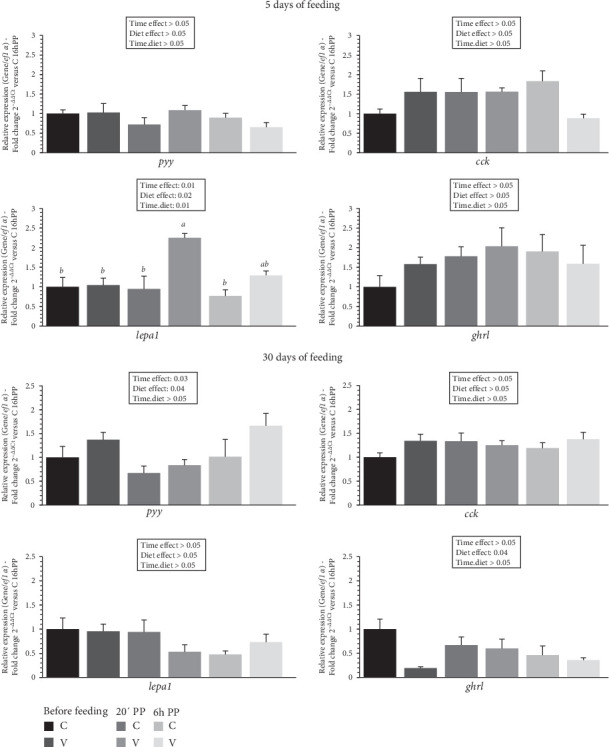
Levels of mRNA encoding genes of intestinal hormones in the gut tissue of trout fed C or V diet from first feeding to 5 days and 30 days. Relative gene expression measured by RT-PCR of selected intestinal hormones markers (*cck*, *pyy*, *lepa1*, and *ghrl*) genes in gut tissue of trout fed from first feeding to 5 days (top panel) and 30 days (bottom panel) the C or V diet before feeding, 20 min, and 6 h after meal. Data are expressed as group mean ± SEM; fold change 2^–*ΔΔ*Ct^ versus before meal for all genes. Two-way ANOVA following by one-way ANOVA when the interaction is significant, Tukey's post hoc test when appropriate; different letters indicate a significant difference (*p*  < 0.05) between diet and time (*n* = 8).

All statistical analyses were performed using R [39] software (version 3.6.1; R Core Team). The tank was the experimental unit for data on growth parameters and Welch Two Sample *t*-test was used for statistical analysis of growth parameters. In comparison, individual fish were the experimental unit for data on gene expression and indolamine/catecholamine release. The results were calculated as means ± 1 standard error of the mean (SEM). Analyses were carried out on untransformed data as criteria for normality of distribution and homogeneity of variances were fulfilled (Shapiro–Wilk and Levene's test, respectively). The interaction between the two factors, diets and time, was assessed using a two-way analysis of variance (ANOVA). When a significant interaction was observed, a one-way analysis of variance (ANOVA) was conducted to assess the individual effects of time after feeding between the two diets. This allowed us to specifically examine the impact of each factor in isolation and determine their respective contributions to the observed results. When appropriate, analysis was followed by Tukey's post hoc test. All statistical analyses were presented in supplemental information 1–6 and selected results in Figures 2–9. Differences were considered significant at *p*  < 0.05.

## 3. Results

### 3.1. Husbandry Parameters

Figure 1 shows the body weight, mortality rates, food intake, and feed efficiency of rainbow trout fry fed either C diet or a completely V diet, during a period of 10 days from the first feeding to 30 days. For the first period from first feeding to 10 days, food intake was significantly lower (−15%) for fish fed the V diet. For the second period measured (11 to 20 days), body weight gain was significantly lower (−14.9%) for fish fed the V diet. For the third period measured (21 to 30 days), body weight gain and food intake were significantly lower (−27.4% and −8.9%, respectively) for fish fed the V diet. The survival rate and feed efficiency did not differ between alevins fed the C versus the V diet for the three periods measured.

### 3.2. Influence of the V Diet on Changes on Fatty Acid Receptor Gene Expression in the Tongue of Trout During the First Month of Feeding.

The mRNA levels of selected fatty acid receptor (*ffar1*, *ffar2a2*, *ffar2b2a*, *ffar2b2b2*, *cd36*, *gpr84*, *fatp1*, *fatp4a*) in the tongue of trout before meal (16 h postprandial), 20 min and 6 h after the meal are presented in Figure 2.

After 5 days of feeding, the mRNA levels of all selected fatty acid receptors genes varied (downregulation) after meal (timing effect). Whereas the selected free fatty acid receptors (*ffar1*, *ffar2a2*, *ffar2b2a*, and *ffar2b2b2*) did not vary with diet proposed, the mRNA levels of *gpr84* were up-regulated for fish fed the V diet.

When comparing the two diets, the mRNA level of *cd36* gene was not significantly affected by the proposed diet. However, a significant interaction (diet × time) was observed, showing an upregulation before meal and at 6 after feeding in fish fed the V diet, compared to V diet 20 minutes and 6 h after meal. A significant interaction (diet × time) was also observed for mRNA level of *fatp1* gene which was upregulated before meal in fish fed the V diet compared to V diet 20 min and 6 h after meal and 20 min after meal for the C diet. The mRNA level of *fatp4a* gene was upregulated 6 h after feeding in fish fed the C diet compared to C and V diet 20 min and 6 h after meal respectively.

After 30 days of feeding, the mRNA levels of all selected ffar genes (*ffar1*, *ffar2a2*, *ffar2b2a*, and *ffar2b2b2*) and *fatp1* were impacted by diet (diet effect, upregulation for fish fed C diet) and after meal (timing effect, downregulation) for *ffar2b2a*.

When comparing the two diets, the mRNA level of *ffar1* genes revealed a significant interaction (diet × time) showing an upregulation before meal in fish fed the C diet compared to V diet before meal. A significant interaction (diet × time) was also observed for mRNA level of *ffar2a2*, *ffar2b2a*, and *ffar2b2b2* genes which were upregulated 20 min after feeding in fish fed the C diet compared to the C diet 6 h after the meal and for the three timing point for the V diet (before meal, 20 min and 6 h after feeding). The mRNA level of *gpr84* gene was upregulated 20 min after feeding in fish fed the C diet compared to V diet and to C diet before meal and 6 h after meal respectively.

### 3.3. Influence of the V Diet on Changes on Amino Acid Receptor Gene Expression in the Tongue of Trout During the First Month of Feeding.

The mRNA levels of selected amino acid receptors (*gprc6a*, *lpar5*, *t1r1-1*, *t1r1-2*, *t1r2-a* and *t1r3*) in the tongue of trout before meal (16 h postprandial), 20 min and 6 h after meal are presented in Figure 3.

After 5 days of feeding, the mRNA levels of all selected amino acid receptor genes varied (downregulation) after meal (timing effect) and by diet (C diet; upregulation) for *lpar5*, *t1r1-1*, *t1r2-a* and *t1r3*.

After 30 days of feeding, the mRNA levels of all selected amino acid receptor genes varied depending on the proposed diet (by C diet; upregulation) and after meal (timing effect, downregulation) for *lpar5*, *t1r1-1*, *t1r2-a* and *t1r3*. When comparing the two diets, the mRNA level of *lpar5* and *t1r1-3* gene revealed a significant interaction (diet.time) showing an upregulation 20 min after feeding in fish fed the C diet compared to all other timings. A significant interaction (diet × time) was also observed for the mRNA level of the *t1r1-1*and *t1r1-2* gene which were upregulated 20 min after feeding in fish fed the C diet, compared to the C diet 6 h after feeding, and before feeding, as well as 20 min and 6 h after feeding for the V diet.

### 3.4. Influence of the V Diet on Changes on Flavor Receptors Gene Expression in the Tongue of Trout During the First Month of Feeding.

The mRNA levels of selected flavor receptors *tasr2a4* (bitter), *otop1* (sour), *grm4a* (umami), and *asic1a* (acid) in the tongue of trout before meal (16 h postprandial), 20 min, and 6 h after meal are presented in Figure 4.

After 5 days of feeding, the mRNA levels of selected flavor receptor genes varied (downregulation) after meal (timing effect) for *tasr2a4*, *asic1a* and *grm4a* and by diet (C diet; upregulation) for *grm4a*. When comparing the two diets, the mRNA level of *otop1* gene revealed a significant interaction (diet × time) showing an upregulation 20 min after feeding in fish fed the C diet compared to C diet before meal and for V diet 20 min and 6 h after feeding. The mRNA level of *asic1a* gene also revealed a significant interaction (diet × time) showing an upregulation before feeding in fish fed the V diet compared to C diet before meal and 6 h after feeding for the C and the V diet. After 30 days of feeding, the mRNA levels for all selected flavor receptor genes varied by the diet (C diet; upregulation) and after meal (timing effect, downregulation) for *otop1*. When comparing the two diets, the mRNA level of *tasr2a4* and *asic1a* gene revealed a significant interaction (diet × time) showing an upregulation 20 min after feeding in fish fed the C diet compared to all other timings for *tasr2a4* (except before meal for C diet) and *asic1a*.

### 3.5. Influence of the V Diet on Changes on Calcium-Signaling Pathway Gene Expression in the Tongue of Trout During the First Month of Feeding.

The mRNA levels selected calcium-signaling pathway markers (*cpla2*, *plcb4*, *iptr3*, *calhm1*, *orai1a*, *orai1b*, *stim1a*, *stim1b*) in the tongue of trout before meal (16 h postprandial), 20 min and 6 h after meal are presented in Figure 5.

After 5 days of feeding, the mRNA levels of selected calcium-signaling pathway markers genes varied (downregulation) after meal (timing effect) for *cpla2*, *plcb4*, *iptr3*, *calhm1*, *orai1a*, *stim1b* and by diet (C diet; upregulation) for *cpla2*, *plcb4* and *stim1a*. When comparing the two diets, the mRNA level of *itpr3* gene revealed a significant interaction (diet × time) showing an upregulation before meal in fish fed the V diet compared to those fed the C diet. Additionally, mRNA levels were higher in fish fed the V diet before meal compared to 2 h and 6 h after feeding. The mRNA level of *calmh1* gene also revealed a significant interaction (diet × time) showing an upregulation before feeding in fish fed the V and the C diet before feeding and 6 h after feeding for V diet compared to the C diet 20 min and for the V diet 20 min and 6 h after feeding. The mRNA level of *stim1b* gene revealed a significant interaction (diet × time) showing an upregulation before feeding in fish fed the V diet compared to V diet 6 h after feeding.

After 30 days of feeding, the mRNA levels of selected calcium-signaling pathway markers genes varied by diet (C diet; upregulation) for *cpla2* and *calmh1*. When comparing the two diets, the mRNA level of *cpla2* gene revealed a significant interaction (diet × time) showing an upregulation before meal in fish fed the C diet compared to the V diet before meal. The mRNA level of *calmh1* gene revealed also a significant interaction (diet × time) showing an upregulation before meal and 20 min after feeding in fish fed the C diet compared before meal for fish fed the V diet.

### 3.6. Indolamine and Catecholamine Metabolites Quantification in Tongue Tissue

The metabolite levels of 5-HT indolamine pathway and dopamine catecholamine pathway turnover in the tongue of trout before meal (16 h postprandial), 20 min, and 6 h after meal are presented in Figures 6 and 7.

For indolamine pathway metabolites (Figure 6) 5 days after feeding, 5-HT levels did not differ after meal but were influenced by the proposed diet (higher levels quantified for C diet). For 5-HIAA, the direct degradation product of 5-HT, the metabolite level did not differ after meal but were influenced by the proposed diet (higher levels quantified for V diet). The ratio 5-HIAA/5-HT ratio was also not different after meal (timing effect) but lower values were quantified for the C diet at all timing.

For indolamine pathway metabolites 30 days after feeding, the 5-HT level did not differ after meal or depending on diet proposed. For 5-HIAA, the metabolite level was higher after meal (timing effect) but lower for the C diet at all timings. When comparing the two diets, fish fed the V diet had a higher 5-HIAA level than fish fed the V diet before meal and also for all timing for the C diet. The ratio 5-HIAA/5-HT ratio was also higher after meal (timing effect) but lower for the C diet for all timing. When comparing the two diets, fish fed the V diet had also a higher 5-HIAA level than fish fed the V diet before meal and also for all timing for the C diet.

For catecholamine pathway metabolites (Figure 7) 5 days after feeding, the L-DOPA (dopamine precursor) was increased by the V diet (diet effect). For HVA, one of the direct degradation products of dopamine, the metabolite level was higher for the C diet (diet effect). The HVA/L-DOPA ratio was lower for fish fed the V diet (diet effect).

For catecholamine pathway metabolites 30 days after feeding, the L-DOPA was increased by the C diet (diet effect). When comparing the two diets, fish fed the C diet had higher L-DOPA 20 min after feeding compared to fish fed the C diet before meal and 6 h after meal, and all timing for the C diet was higher compared to fish fed the V diet for the three timing. For HVA, the metabolite level was not different depending of diet proposed and after meal. The HVA/L-DOPA ratio was lower for fish fed the C diet (diet effect).

### 3.7. Neuropeptide Gene Expression Profile in Brain Tissue

The mRNA levels of selective neuropeptide gene in the brain of trout before meal (16 h postprandial), 20 min, and 6 h after meal are presented in Figure 8.

For selected orexigenic markers 5 days after feeding, the mRNA level of *agrp1* was upregulated after meal (timing effect). For anorexigenic markers, the mRNA level of *pomcb* was upregulated after meal (timing effect).

For selected orexigenic markers 30 days after feeding, the mRNA level of *agrp1* was upregulated by the C diet (diet effect). When comparing the two diets, the mRNA level of *npya* gene revealed a significant interaction (diet × time) showing an upregulation 20 min after feeding for fish fed the V diet compared to C diet 20 min after feeding. For anorexigenic markers, the mRNA level of *cartpt* was upregulated by the C diet (diet effect). When comparing the two diets, the mRNA level of *pomcb* gene revealed a significant interaction (diet × time) showing an upregulation 20 min after feeding for fish fed the C diet and 6 h after feeding for C and V diet compared to the C diet and the V diet before feeding and 20 min after feeding for the C diet. The mRNA level of *cartpt* gene also revealed a significant interaction (diet × time) showing an upregulation before feeding for fish fed the C diet compared to V diet before feeding and 20 min after feeding.

### 3.8. Intestinal Hormone Gene Expression Profile in Gut Tissue of Trout

The mRNA levels of selected intestinal hormones gene in the gut of trout before meal (16 h postprandial), 20 min and 6 h after meal are presented in Figure 9.

After 5 days of feeding, the mRNA levels of selected intestinal hormone genes varied after meal (timing effect) and by diet (V diet; upregulation) for *lepa1*. When comparing the two diets, the mRNA level of *lepa1* gene revealed a significant interaction (diet × time) showing an upregulation 20 min after feeding in fish fed the V diet compared to the C and the V diet before meal and 6 h after feeding for the C diet.

After 30 days of feeding, the mRNA levels of selected intestinal hormone genes varied after meal (timing effect) for *pyy* and by diet for *pyy* (V diet; upregulation) and *ghrl* (C diet; upregulation).

## 4. Discussion

During the first month of feeding, our data reveal a significant reduction in weight gain in fish fed a V diet starting from the second period (days 11–20) and continuing until the end of the feeding experiment (days 21–30). These findings align with our previous study, which showed reduced final body weight after 20 and 41 days of feeding on a plant-based diet [5]. In contrast, Lazzarotto et al. [4] did not observe any weight differences in fish during the first month of life. Interestingly, while this other study reported a significant drop in survival, up to 35% after 9 weeks, starting from the first month, we observed no such effect on survival in our current or previous experiments. One possible explanation for these discrepancies lies in the analysis of two key parameters that influence mortality and growth: feed intake and feed efficiency. In our study, and for the first time, we observed a reduction in feed intake during both the first feeding period (from the initial meal until day 10) and the second period (days 21–30), with no effect on feed efficiency. These results are consistent with our earlier findings, which also showed a reduction in cumulative feed intake after 20 and 41 days but no impact on feed efficiency [5]. The older study by Lazzarotto et al. [4] did not investigate feed intake or efficiency, though it strongly suggested that reduced feed intake was a major factor contributing to the decreased survival of fish fed a plant-based diet. The survival differences observed between these studies may be partially explained by the time lapse between the experiments. The study conducted by Lazzarotto and colleagues took place a decade ago, while the studies by Baranek et al. [5] and our current research are more recent, conducted 3 and 1 years ago, respectively. Indeed, the nearly 10-year gap between studies likely accounts for the improved acceptance of plant-based diets by alevins today. Advances in plant oils and raw materials have reduced antinutritional factors, enhancing feed quality and performance in farmed fish [1, 40]. In their study, Lazzarotto et al. hypothesized that the 35% mortality rate in fish fed a plant-based diet was due to the depletion of yolk reserves, leading to starvation after 9 to 12 weeks. They attributed this to a severe refusal to feed. While we did observe a reduction in feed intake in our study, it was mild, and since no survival differences were noted between the diets, we believe that the fish were feeding but consuming less, which ultimately impacted their growth rather than causing mortality. A key factor explaining the survival differences between the studies likely lies in the composition of the diets. As noted in FAO reports [1, 40], continuous improvements in the quality of plants ingredients for FM and FO have made plant-based diets more acceptable to fish over time. The almost 10-year gap between studies likely explains why plant-based diets are now better accepted by alevins than they were in the past. Notable advancements include improvements in the quality of plant oils and raw materials, resulting in feeds with fewer antinutritional factors or toxic compounds and better overall performance for farmed fish [1, 40].

Our data suggest that a reduction in feed intake may explain the growth deficit observed after 1 month, and potentially over the long term, as seen in our previous studies [4, 5, 41, 42]. By examining the gustatory (tongue) sensory systems pathways primarily involved in regulating feed intake, it is possible to establish a connection between reduced feed intake and impaired growth. The first pathway relates to nutrient receptors. Our data show that the fatty acid receptors of the FFAR type, selected in this study and recently characterized at the genome level of trout through phylogenetic and syntenic analysis [37] tissue distribution [9], as well as their postprandial regulation [10], are regulated by meal intake within the first 5 days after the initial feeding. However, they do not appear to be influenced by the type of diet (C versus V) provided during this period. These changes after 30 days of feeding, where we observe minimal gene expression variation in fish fed the V diet, contrast with the receptors, which appear far more responsive in the tongues of trout fed the C diet. Interestingly, the data at 5 days align with our previous findings at 8 months [10], where *ffar*s were downregulated after a meal and seemed more sensitive to the V diet. Conversely, in this study, they appear more responsive to the commercial-like diet at 30 days. When examining other fatty acid receptors selected genes in this study (*cd36*, *gpr84*, *fatp1a*, and *fatp4*), we observed an opposite pattern, with heightened sensitivity to both diet and meal after 5 days of feeding, but minimal effects after 30 days. These findings, consistent with various studies investigating these receptors across different tissues [25, 43–46], highlight the fine-tuned sensitivity of fat taste receptors in rainbow trout. One possible explanation for these differences lies in the varying composition of fatty acids in the diets, which differ significantly across several fatty acid types. The large number of fat receptors and their distinct affinities for specific nutrients as well known in mammals [47], which remain largely undetermined in farmed fish, may further explain the differential response between receptors over time.

In mammals, the sweet and especially TAS1Rs taste receptors have been reported to be highly sensitive to nutrients such as amino acids [48]. In fish, a few studies have been carried out on the taste receptor system in grass carp (*Ctenopharyngodon idella*) and mandarin fish (*Synchiropus splendidus*), mainly focusing on the expression of TAS1Rs [49, 50]. In trout, mRNAs encoding *t1r* have been described in the gut [51, 52]. As for mRNAs encoding other amino acid receptors, such as *lpar5* and *gprc6a*, they have also been detected in different regions of the gut of Atlantic salmon (*Salmo salar*) [53] and rainbow trout [52]. However, no data currently exist on the fish tongue system, despite its potential to offer important insights into how feeding habits develop when exposed to plant diets, such as the V diet used in our study. This knowledge could be key to improving the acceptance and effectiveness of alternative feeds to fish meal and fish oil in aquaculture. Here, our data show that amino acid and sugar receptors are rapidly regulated after just 5 days of feeding, both by the meal and in fish fed the C diet. These effects on the development of tongue receptor responses persist at 30 days, with an even greater sensitivity observed in the taste receptors of trout fed the C diet compared to the V diet. These interesting findings may explain the reduced feed intake observed in fish fed the V diet. Regarding the amino acid composition of the diet, the most significant differences between the C and V diets are primarily limited to a few amino acids. Specifically, glycine is 34% higher in the C diet compared to the V diet. In fish studies, while 0.5% glycine did not affect feed intake or growth performance in grass carp (*C. idella*) or Nile tilapia (*Oreochromis niloticus*) [54], but concentrations of 1%2% have been shown to enhance growth in hybrid striped bass (*Morone saxatilis* ♀ × *Morone chrysops* ♂), with a tendency to increase feed intake [55]. In contrast to glycine, two amino acids, proline and leucine, compensate for this difference in the V diet, with increases of 17% and 16%, respectively. A previous study in rainbow trout [56] demonstrated that intracerebroventricular administration of leucine reduced food intake. Proline is known to have high palatability in carnivorous fish species [57], including rainbow trout, and has been identified as the most potent amino acid to elicit gustatory responses in both electrophysiological [27] and behavioral studies [58]. However, regarding its impact on food intake, intraperitoneal injection of proline did not influence the food intake of rainbow trout [59]. More recently, oral administration of proline to juvenile rainbow trout significantly reduced voluntary food intake at 2 and 3 h post-administration [26]. This effect is consistent with an increased anorexigenic potential in the hypothalamus, demonstrating that gustatory detection of proline triggers a satiety signal that regulates food intake, ultimately leading to a reduction in feeding. Although further studies are needed to confirm these findings in alevins, it is reasonable to speculate that amino acid detection mechanisms in alevins could have similar effects as observed in juvenile trout, with proline inducing an anorexigenic response and glycine exerting an orexigenic effect. In connection with this, in the brains of the alevin trout at 30 days, the anorexigenic marker *pomcB* was upregulated 20 min after feeding in trout fed the V diet, while the orexigenic marker *cartpt* was higher in the brains of trout fed the C diet. To further explore these mechanisms, it would be valuable to perform oral amino acid administration studies to investigate their impact on the regulation of neuropeptides in the trout's brain, particularly in relation to food intake. Regarding flavor taste receptors, our data at 30 days show an increased sensitivity across all the receptors studied, with an upregulation in trout fed the C diet. It is well-established that fish can detect the five basic tastes amino acids (umami), sugars (sweet), bitter compounds (bitter), salts (salty), and acids (sour) [14]. Both attractive and aversive taste responses in fish have been well documented, with some substances (*e.g*., quaternary amines, nucleotides, organic acids) preferred, while others (e.g., quinine hydrochloride, caffeine) elicit aversive behaviors. Sucrose is a notable case, often perceived as neutral or deterrent in species including channel catfish (*Ictalurus punctatus*) [60] or kutum (*Rutilus frisii kutum*) [61] due to its low presence in aquatic ecosystems and minimal dietary value for fish, especially carnivores [62, 63]. Herbivorous or omnivorous fish, whose diets consist mainly of algae, tend to find sucrose more palatable [14]. Interestingly, some carnivorous species, like rainbow trout [64], do show positive responses to sugars, though reactions can vary depending on concentration and species-specific dietary needs. Our data suggest that flavors in the diet may play a role in regulating food intake, particularly through the tongue sensory detection system. Further studies on the effects of flavors in trout and other farmed fish at the alevin stage would be necessary to investigate this hypothesis in greater detail.

In mammals [65], as well as in fish species such as medaka (*Oryzias latipes*) [66], zebrafish (*Danio rerio*) [67], and rainbow trout [10, 25] the vast majority of taste receptors, whether for nutrients or flavors, are G-protein-coupled receptors (GPCRs) containing a G*α* subunit. Activation of these receptors triggers intracellular calcium signaling, which is essential for transmitting the tongue sensing signal. Although our data show that the calcium signaling pathway is well-developed and regulated by the meal both at 5 and 30 days of feeding, some markers appear more sensitive in trout fed the C diet. This could indicate a finer regulation of upstream receptors, as previously discussed. However, more specific studies, particularly at the protein level or using advanced techniques such as, real-time calcium activity imaging via microscopy, are necessary to determine whether certain diets can disrupt this calcium signaling pathway in fish.

The results of calcium accumulation within the cell lead to membrane depolarization, which in turn culminates in the release of neurotransmitters on the basolateral side of taste cells, where synapses are formed with afferent taste nerve fibers [68, 69]. The signal is then relayed to the nucleus of the solitary tract in the medulla oblongata through gustatory nerves (VII and IX) [70]. Once at the central level, this gustatory signal from the tongue is processed and plays a crucial role in regulating feeding behavior [71]. In rainbow trout, we recently demonstrated the presence of serotonin in the tongues of juveniles, as well as its fine regulation by both meals [10] and the diet provided [5]. In this study, we confirmed similar findings in the tongues of alevins as early as their first month of life, showing a similar sensitivity to both meal timing and the diet offered. As in our studies on juveniles, a significant impact is observed in alevins fed a V diet, which appears linked to a marked degradation of serotonin. These results are further supported by the 5-HIAA/serotonin ratio, which reflects serotonin turnover [72]. Although this effect manifests more rapidly than changes in calcium signaling, it is consistent with findings in mammals, suggesting a link between calcium signaling pathways and serotonin release in taste bud cells of mice and humans [73]. As previously mentioned, these findings may help explain the observed changes in food-related neuropeptides in the brain, potentially due to impaired activation of the tongue-brain axis. This disruption could lead to stronger regulation of both anorexigenic and orexigenic genes, as revealed in our study for fish fed the V diet, ultimately impacting food intake and the growth of our alevins. In addition to our data on indolamines turnover, and to our knowledge, for the first time in fish (and rarely studied in mammals) [74], we quantified and revealed the presence of catecholamines, specifically L-DOPA and its degradation product HVA, in the tongues of alevins. A rare study published over 30 years ago on the effects of diet revealed that dopamine levels in the brain (telencephalon, hypothalamus, and mesencephalon) were not influenced by different dietary regimes (commercial, marine, or plant-based diet) [75]. The findings suggested that after 5 months of feeding, the lack of variation observed could be explained by the trout's (*Salmo gairdneri*) adaptation to the diets. This study already hints that brain tissue might not be the most relevant marker for detecting subtle variations, such as those induced by diet, compared to more significant environmental stressors, such as hypoxia, as investigated in this study or, more recently in trout [76] and other species under various stress conditions [77, 78]. Moreover, in the tongue, while food intake did not appear to influence L-DOPA and HVA levels, the diet did have an impact on catecholamine turnover. After just 5 days of feeding, we observed that dopamine pathway activity (as indicated by the HVA/L-DOPA ratio) was significantly higher in fish fed the plant-based V diet. However, after 30 days, this trend completely reversed, favoring those on the commercial diet. Although the implications of these effects are not yet fully understood, as no previous studies have observed this in fish, it is evident that the dopamine pathway, measured through L-DOPA and HVA, could serve as an important tongue sensing early marker depending on the diet in fish farmed. One possible hypothesis is that the increased dopamine pathway activity after 5 days might be linked to a compensatory mechanism for impaired food detection. This heightened activity seems to diminish and become dysregulated after 30 days, similar to serotonin, potentially leading to a deficiency in food detection signaling to the brain, ultimately affecting both food intake and growth performance.

Intestinal hormones are now well-recognized as secondary regulators of food intake in mammals [79] and also in fish [57], such as trout [31]. Around mealtime (before and after ingestion), food reaches the stomach and then the intestine, depending on the species. At this stage, the secretion of orexigenic and anorexigenic hormones regulates digestion by influencing intestinal motility, the secretion of digestive peptides, and, importantly, the control of hunger through the gut-brain axis [80]. In our study, while 5 days of feeding appeared to have minimal impact on hormonal regulation, data after 30 days suggest that these hormones could play a significant role as regulators of food intake. The expression of *pyy*, an anorexigenic hormone, was upregulated in animals consuming the V diet, whereas the opposite was observed for the orexigenic hormone ghrelin (*ghrl* gene). These findings align with food intake data, strongly suggesting that as early as the first month of feeding, these hormones act as key regulators of both food intake and feed efficiency, playing a crucial role in this regulatory process. Studying the specific role of these intestinal hormones during the alevin stage would be particularly interesting to clarify their precise impact on food intake compared to their influence on feed efficiency and its relationship with alevin growth on plant-based diet.

## 5. Conclusion

In conclusion, our study highlights the early and heightened sensitivity of the tongue sensory system in rainbow trout alevins to detect plant diets devoid of FM/FO. This novel food source triggers distinct regulatory responses that influence feed intake and growth, altering the expression of genes involved in fatty acid, amino acid, sweet, and flavor receptors, as well as calcium signaling pathways. Additionally, early changes in serotonin and dopamine turnover suggest that these pathways play a crucial role in integrating brain responses to feeding. These regulatory shifts underscore how significant nutritional variations can disrupt feeding behavior from the first meal, impacting food detection and diet acceptance. Future research should focus on optimizing plant-based diets to support early growth and health, advancing sustainable aquaculture practices. Specifically, testing key nutrients such as amino acids, sugars, fatty acids, and flavors at early or later stages could help refine feed formulations, paving the way for precision nutrition in aquaculture.

## Figures and Tables

**Table 1:** Ingredients and composition of the experimental diets.

Ingredient (%)	Diet	
	Commercial-like (C) diet	Plant-based (V) diet
Fish meal	32.0	0.0
Soybean meal	9.0	23.3
Peas lysamine protein concentrate	17.7	25.5
Corn gluten	11.9	25.6
Extruded whole wheat	8.6	2.0
Gelatinized wheat starch	2.2	0.0
Sunflower lecithin	0.0	3.0
L-lysine	0.0	1.0
L-methionine	0.4	1.0
CaHPO_4_·2H_2_O	0.0	1.5
Mineral premix^a^	1.5	1.5
Vitamin premix^b^	1.5	1.5

Fish oil	11.40	0.0
Rapeseed oil	3.80	1.80
Palm oil	0.0	8.30
Linseed oil	0.0	4.00

Composition (% of dry matter, DM)		
Dry matter (in % of diet)	94.47	96.03
Crude protein	54.54	52.29
Crude lipid	17.03	20.61
Ash	9.96	7.24
Energy (kJ.g^−1^)	24.37	24.33

^a^Mineral premix: (g or mg kg^−1^ diet): calcium carbonate (40% Ca), 2.15 g; magnesium oxide (60% Mg), 1.24 g; ferric citrate, 0.2 g; potassium iodide (75% I), 0.4 mg; zinc sulphate (36% Zn), 0.4 g; copper sulphate (25% Cu), 0.3 g; manganese sulphate (33% Mn), 0.3 g; dibasic calcium phosphate (20% Ca, 18% P), 5 g; cobalt sulphate, 2 mg; sodium selenite (30% Se), 3 mg; KCl, 0.9 g; NaCl, 0.4 g (UPAE, INRAE).

^b^Vitamin premix (IU or mg kg^−1^ diet): retinol acetate, 55,000 IU; cholecalciferol, 2500 IU; DL-*α*-tocopherol acetate, 50 IU; sodium menadione bisulfate, 10 mg; thiamin-HCl, 1 mg; riboflavin, 4 mg; niacin, 10 mg; D-calcium pantothenate, 20 mg; pyridoxine-HCl, 3 mg; D-biotin, 0.2 mg; folic acid, 1 mg; cyanocobalamin, 10 μg; L-ascorbyl-2-polyphosphate, 50 mg; myo-inositol, 0.3 g; choline, 1 g (UPAE, INRAE).

**Table 2:** Fatty acid composition of experimental diets (% of total fatty acids).

	Diet	
	Commercial-like (C) diet	Plant-based (V) diet
C14:0	4.8	0.3
C15:0	0.5	0.1
C16:0	15.7	15.5
C17:0	0.5	0.1
C18:0	3.4	3.9
C20:0	0.3	0.3
C22:0	0.0	0.2
C24:0	0.0	0.0
Sum of saturated fatty acids	**25.1**	**20.3**
C16:1 *ω*-7	5.7	0.2
C18:1 *ω*-9	27.6	28.8
C20:1 *ω*-9	2.2	0.3
C22:1 *ω*-9	1.6	0.0
Sum of MUFAs	**37.1**	**29.2**
C18:2 *ω*-6 (LA)	10.5	23.8
C18:3 *ω*-6	0.2	0.0
C20:2 *ω*-6	0.1	0.0
C20:4 *ω*-6 (ARA)	0.7	0.0
Sum of *ω*-6 LC-PUFAs	**11.6**	**23.8**
C16:4 *ω*-3	1.2	0.0
C18:3 *ω*-3 (ALA)	3.7	26.6
C18:4 *ω*-3	1.5	0.0
C20:4 *ω*-3	0.3	0.0
C20:5 *ω*-3 (EPA)	6.4	0.0
C21:5 *ω*-3	0.1	0.0
C22:5 *ω*-3	0.8	0.0
C22:6 *ω*-3 (DHA)	9.4	0.0
Sum of *ω*-3 LC-PUFAs	***23.8***	***26.6***
Sum of *ω*-3 (EPA + DHA)	***15.8***	***0.0***
*ω*-3 (DHA + EPA)/*ω*-6	***1.4***	***0.0***

*Note*: Bold values indicate the addition to the sum of saturated fatty acid quantified.

**Table 3:** Amino acid composition of experimental diets (% of dry matter).

Amino acids	Commercial-like (C) diet	Plant-based (V) diet
Alanine	2.84	2.67
Arginine	3.12	3.06
Aspartatic acid	4.43	4.18
Cysteine	0.52	0.61
Glutamatic acid	7.87	8.76
Glycine	2.23	1.66
Histidine	1.16	1.1
Isoleucine	2.3	2.3
Leucine	4.33	5.01
Lysine	3.14	3.21
Methionine	1.43	1.71
Phenylalanine	2.34	2.61
Proline	2.5	2.92
Serine	2.16	2.3
Threonine	1.91	1.72
Tryptophane	0.47	0.42
Tyrosine	1.66	1.8
Valine	2.45	2.29

**Table 4:** Nucleotide sequences of the PCR primers used to evaluate mRNA abundance of transcripts by real-time quantitative PCR.

Transcript	Forward primer	Reverse primer	Accession number
Reference			
* eef1a1*	TCCTCTTGGTCGTTTCGCTG	ACCCGAGGGACATCCTGTG	ENSOMYG00000038328
* krt8b*	TGGCTACTCCAGTGGTTTCG	CCGCTACCGGAGCTGTAGTT	ENSOMYG00000057091
Fatty acid receptors			
* ffar1*	ACTGTTGCACCTGAGTCTGG	GCTGGTCCTGGGTGAAGTTC	ENSOMYG00000041396
* ffar2a2*	GACAACTTCACCCAGGAGCA	AGCAGAACACACAGGCCAG	ENSOMYG00000030315
* ffar2b2a*	CCCATCCAACACTCGCTGAA	TGATGACGACGATGCTCAGG	ENSOMYG00000030493
* ffar2b2b2*	GTCCAGTACCATCAACGCCA	CTGCACACTCTCCAACAGGGT	ENSOMYG00000005604
* cd36*	CCTGCTCTCCAAAATCCACG	TATAGTCCCGTTCGCCAGTC	ENSOMYG00000030873
* slc27a1*	GGGCTACATGTACTTCCGGG	CCAGCAGACCACTCAGTGTT	ENSOMYG00000012230
* slc27a4a*	GAAACATTGTTGGCGTGGGG	TCGTGCAGTTGTACTTGGCA	ENSOMYT00000042886.1
* gpr84*	CTCCTCCACCACCTCTTCAG	TCAGCAATGTTCAGCAGCAG	ENSOMYG00000019195
Amino acid/sweet receptors			
* gprc6a*	AGTGCAAGTTCCCCTCGTTT	CGTCACTGCCTATCACTCCC	ENSOMYG00000014183
* lpar5*	TGTGGCTGCTTGTGATCAAC	GGGGAACTGGGATTGAAGGA	ENSOMYG00000007169
* t1r1-1*	GCTGGTGCGTTTTAACTGGA	GGCGATGCAGATGTCAAAGT	ENSOMYG00000023953
* t1r1-2*	ATCCCTCATTCCTGCGAACC	TCATCACCGCTGCTGACAAA	ENSOMYG00000021237
* t1r2-a*	CTGTCGCCATCACACTCATG	GCAGACTTAACAACCGGCTT	ENSOMYG00000021237
* t1r3*	CCAGATACAGAGCCAACCCA	GACCCTGAAAGCTGACCTCT	ENSOMYG00000012034
Flavor receptors			
* tasr2r4*	TGTTTTCCTGCACCTCTCCA	ACTGCTCAGCCACACATACA	ENSOMYG00000012237
* otop1*	GAGACCCCGAGCAAGAAGAG	GTTCATGGGCACGGCAAAAT	ENSOMYG00000046532
* asic1a*	GAGCAGCTCTGAGTCTGGAG	TGTGCTGGATGGTGAGGAAG	ENSOMYG00000062884
* grm4a*	CCATTTCATCTGGGTGGGCT	CCTCTGATGGACTGGCGTTT	ENSOMYG00000030638
Calcium signaling pathways			
* cpla2a*	CGTGGCTGGACTTTCTGGAT	GCTCACACACCTCATCAGCT	ENSOMYT00000110702.1
* plcb4*	CCGAGGCGTCTGAGGATAAC	GTAGGGGTGGATGTTGGTGG	ENSOMYG00000018058
* itpr3*	TACCTGCGACTACTCTGCCT	AATGATGATTGCAGGGCCCA	ENSOMYT00000051670.1
* stim1a*	GAACCTTGACCCCAGCATCA	GGAGATTGCAGAGTACCGGG	ENSOMYG00000038250
* stim1b*	CGGACGTGTACAACTGGACA	CCTTCCGTCCAGATTGTGCT	ENSOMYG00000011142
* orai1a*	CTATGGTGGAGGTGCAGCTG	TCATCAGGGCGAAAAGGTGC	ENSOMYG00000005009
* orai1b*	TGAAGGCTTCCAGTCGAACG	GGCACTGAAGGCAATGAGGA	ENSOMYT00000069145
* calmh1*	CTACGGGATTGGACTGCTGG	TCTTCCCCGTCGGTCTTTTC	ENSOMYT00000065284.1
*Neuropeptide*			
* npya*	AAGGCAGAGGTGAGTGCTGT	AGCCTGTGGCTCACTAATC AA	ENSOMYG00000006572
* agrp1*	ACCAGCAGTCCTGTCTGGGTAA	AGTAGCAGATGGAGCCGAACA	ENSOMYG00000068541
* pomca*	ACAAGATGAACCACTTCCGCT	TGAACAACGTGAGCAGTGGT	ENSOMYG00000014502
* cartpt*	ACCATGGAGAGCTCCAG	GCGCACTGCTCTCCAA	ENSOMYG00000036163
*Intestinal hormone*			
* cck*	GGGTCCCAGCCACAAGATAA	TGGATTTAGTGGTGGTGCGT	ENSOMYT00000059759.2
* pyy*	GGCTCCCGAAGAGCTGGCCAAATA	CCTCCTGGGTGGACCTCTTTCCA	ENSOMYT00000096954.2
* lepa1*	TTGCTCAAACCATGGTGATTAGGA	GTCCATGCCCTCGATCAGGTTA	ENSOMYT00000056091
* ghrl*	GGTCCCCTTCACCAGGAAGAC	GGTGATGCCCATCTCAAAAGG	ENSOMYG00000008000

Abbreviations: *agrp* (1), agouti-related peptide; *asic1a*, acid-sensing ion channel 1a; *calhm1*, calcium homeostasis modulator 1; *cartpt*, amphetamine-related transcript; *cck*, cholecystokinin; *cd36*, cluster of differentiation 36; *cpla2a*, Phospholipase A2; *eef1a1*, elongation factor 1*α*; *ffar*, (*1*, *2a2*, *2b2a*, *2b2b2*), free fatty acid; *ghrl*, ghrelin and obestatin prepropeptide; *gmr4* (*a*), glutamate metabotropic receptor 4; *gprc6a*, G protein-coupled receptor family C group 6 member A; *gpr84*, G-protein coupled receptor 84; *itpr3*, inositol 1,4,5-triphosphate receptor 3; *krt8b*, keratin 8b; *lepa1*, leptin a1; *lpar5*, lysophosphatidic acid receptor 5; *npy* (a), neuropeptide Y; *orai* (1a, 1 b), calcium release-activated calcium modulator; *otop1*, otopetrin 1; *plcb* (4), phospholipase C-*β*; *pyy*, peptide YY; *stim* (*1a*, *1 b*), stromal interaction molecule; *pomc* (a), proopiomelanocortin; *slc27a1*, fatty acid transporter 1*; slc27a4* (*a*), fatty acid transporter 4; *t1r* (*t1r1-1*, *t1r1-2*, *t1r2-a*, *t1r3*), taste receptor type 1 (member 1, 2 and 3); *tasr2r4*, taste 2 receptor member 4.

## Data Availability

All data supporting the findings in this study are available within the article. Raw phenotypic datasets are available from the Recherche Data Gouv public repository entitle “ Effects of plant-based diet in first month of feeding of alevin rainbow trout (*Oncorhynchus mykiss*) on gustatory nutrient sensing systems developpement”, https://doi.org/10.57745/789EYO. Correspondence and material requests should be addressed to Jérôme Roy (jerome.roy@inrae.fr).
